# Treatment Algorithm for Management of Benign Prostatic Obstruction: An Overview of Current Techniques

**DOI:** 10.3390/life13102077

**Published:** 2023-10-18

**Authors:** Thomas Hughes, Philip Harper, Bhaskar K. Somani

**Affiliations:** 1 Department of Urology, South Warwickshire University Hospital NHS Foundation Trust, Warwick CV34 5BW, UK; tom.hughes14@nhs.net; 2 Department of Surgery, Guy’s and St Thomas’ NHS Foundation Trust, London SE1 9RT, UK; 3 Department of Urology, University Hospital Southampton NHS Foundation Trust, Southampton SO16 6YD, UK

**Keywords:** BPH, prostate, laser, PAE, MIST, Rezūm, Urolift

## Abstract

The management of benign prostatic obstruction (BPO) should involve a treatment algorithm that takes into account prostate size, and patient’s symptoms and preference with the aim of helping with urinary symptoms and enhance quality of life. The diagnostic assessment for men with lower urinary tract symptoms (LUTS) should be comprehensive to help choose the best management strategy. Strategies from lifestyle modifications to medical treatment with alpha blockers and/or 5-alpha-reductase inhibitors to surgical procedures can all be used in the management algorithm. Surgical management ranges from transurethral resection of prostate (TURP) to minimally invasive surgical therapies (MIST) including laser therapies such as Holmium laser enucleation (HoLEP) and photoselective vaporisation (PVP), aquablation, Rezūm system, prostate artery embolisation (PAE), prostatic urethral lift (PUL), temporary implantable nitinol device (iTind) and Optilume BPH catheter system. BPO is a common urological condition that has a significant impact on quality of life and economic burden globally and is likely to become increasingly prevalent with an ageing population. Selecting the most appropriate treatment modality will depend on the individual patient preferences, availability of resources, cost, anatomical factors and the goals of treatment.

## 1. Introduction

Benign prostatic obstruction (BPO) is one of the most common causes of lower urinary tract symptoms (LUTS) in middle-aged and older men. Benign prostatic hyperplasia (BPH) is a histological diagnosis that increases in prevalence with age, and autopsy studies have shown that 80–90% of men will have evidence of BPH in their 70s or 80s [1]. However, not all of these men will develop LUTS with symptoms typically arising when the prostatic enlargement results in obstruction [1]. Epidemiological data suggest a lifetime prevalence of LUTS suggestive of BPO of 26.2% and this increases with age [2]. With a globally ageing population, the incidence of BPO is continuing to increase, which is concerning given the already estimated $73.8 billion annual cost burden [3]. LUTS also have substantial impact on the quality of life (QoL) of patients, with one study reporting that severe LUTS produce a similar impact on QoL to a heart attack or stroke [4]. BPO is a progressive condition and factors associated with clinical progression include increased age, increased prostate volume, elevated PSA and low peak urinary flow rate (Q_max_) [5].

Fortunately, numerous treatments options are available to manage LUTS secondary to BPO including both medical and surgical. Traditionally, transurethral resection of prostate (TURP) has been the gold standard for surgical treatment of BPO although this is not without risk and the potential for sexual dysfunction is unacceptable to some patients [6,7]. Over the last two decades a wide array of different surgical techniques have become available and some of these are developing robust evidence, including the use of holmium laser enucleation of prostate (HoLEP) for larger prostates. There are also a group of minimally invasive surgical treatment (MIST) options for BPO which are often associated with faster post-operative recovery, day surgery suitability and lower risk of sexual dysfunction [8].

Both the European Association of Urologists (EAU) and American Urology Association (AUA) publish guidelines on the management of LUTS secondary to BPO [9,10,11]. BPO management is not a case of ‘one size fits all’ and will depend on many factors including patient preference, goals of treatment, anatomical factors and availability of resources. The purpose of this paper is to review the current management techniques in the treatment of BPO available to the urologist and support decision making.

## 2. Diagnostic Workup

The diagnostic evaluation of a man presenting with LUTS begins with a comprehensive assessment of the patient’s history to elicit the nature of the LUTS and to help exclude other differential diagnoses including urethral stricture, distal ureteric stone, neurogenic bladder dysfunction, urinary tract infection, urinary tract malignancy and detrusor overactivity [9].

Whilst physical examination including digital rectal examination is an integral component of the assessment of such patients, it should be noted that correlation between estimated and actual prostate volume is poor [12]. Three-day frequency volume charts are useful tools to assess urinary function and limit the impact of recall bias [9]. Urinalysis is an inexpensive, readily available test that may suggest alternative causes of LUTS including urinary tract infection (UTI), diabetes mellitus or malignancy, that would require additional investigation [9]. Prostate specific antigen (PSA) should be measured if a diagnosis will change management and patients should be counselled regarding testing and the implications of an elevated test. Renal insufficiency is more common in men with BPO, and renal function should be measured if impairment is suspected or surgery is planned.

Uroflowmetry and post void residual (PVR) are standard investigations in the assessment of LUTS and can be used as follow-up investigations to measure response to treatment. A validated symptom score questionnaire can quantify the severity of LUTS and assess response to treatment over time with the international prostate symptom score (IPSS) being widely used. The eight-item IPSS assesses seven symptoms with an additional question for QoL, and can stratify symptom severity as mild, moderate or severe [13].

Further investigations including ultrasound of the upper urinary tract may be indicated in the presence of haematuria, history of urolithiasis or large post void residual. Imaging of the prostate, typically with transrectal ultrasound, to assess size should be undertaken prior to surgical treatment as this will impact on treatment decisions [9,10]. Cystoscopy is indicated if there is haematuria, suspicion of a urethral stricture or if findings would alter treatment (e.g., a median lobe may limit suitability of certain procedures). In cases of diagnostic uncertainty, urodynamics may be useful prior to surgery but is not necessary in all cases [9].

The initial presentation of some men with BPO may be urinary retention, either acute or chronic. The assessment and work up of these patients will differ slightly, particularly in patients with high pressure chronic retention (HPCR); in this group, the management priorities are protecting renal function rather than symptom management alone [14].

## 3. Management

**Figure 1:**
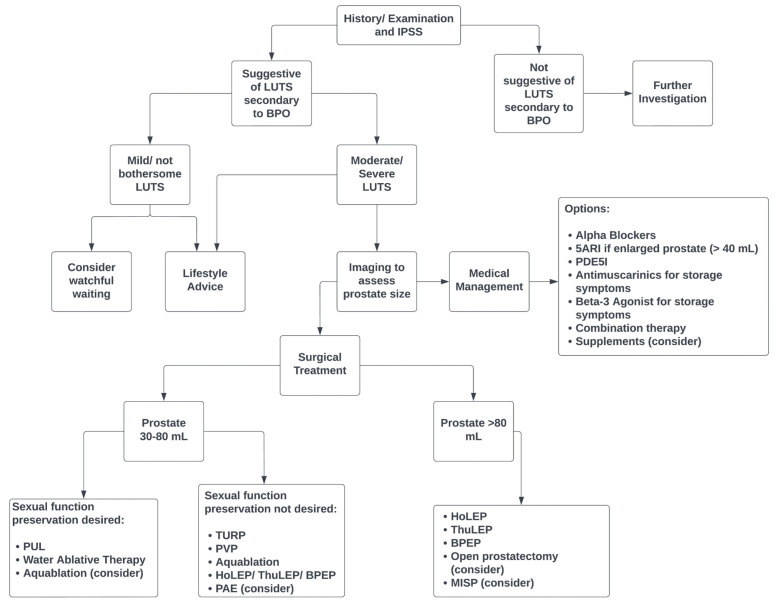
Suggested treatment algorithm for assessment and treatment of male LUTS secondary to BPO.

The management options for LUTS secondary to BPO are broad and the suitability of these options varies between patients (Figure 1). Surgery may not be appropriate for severely frail patients in which the anaesthetic risks are deemed too great. Similarly, medical management would be inappropriate in a patient presenting with HPCR. The risk profile differs for each technique and therefore extensive counselling to enable a shared decision-making process is recommended [7,15].

## 4. Conservative Management

Behavioural and dietary modifications can be simple ways in which patients can experience an improvement in symptoms. Dietary advice includes moderating caffeine and alcohol consumption, reducing intake of fluids at night and altering the timing of diuretic medications to ensure that diuresis is predominately during the daytime [16]. Double-voiding and urethral milking may be helpful for patients with larger residual volumes and post-micturition dribbling, respectively. In some cases, particularly in those with less bothersome symptoms, education and reassurance alone with a watchful waiting approach and regular follow-up may be an appropriate initial management strategy [16].

## 5. Medical Management

### 5.1. Alpha Blockers

Alpha-adrenergic (1a) receptor blockers (ABs) (e.g., alfuzosin, doxazosin, silodosin, terazosin and tamsulosin) have been used to improve LUTS since the 1980s. Alpha-1a adrenoreceptors are found in the smooth musculature of the bladder neck, urethra and prostate [17]. Endogenously released noradrenaline acts on these receptors to increase prostatic tone and therefore bladder outflow resistance [17].

Randomised controlled trials comparing ABs with placebo report that ABs result in a 30–45% improvement in IPSS score, although placebo also results in improvement of symptoms in 10–30% [18]. Both AUA and EAU guidelines do not advocate one AB over another as the clinical efficacy is deemed to be broadly similar [9,10,18]. Additionally, ABs can be used in patients with acute urinary retention (AUR) secondary to BPO to increase the likelihood that future trial without catheter (TWOC) will be successful [19]. Asthenia, dizziness and postural hypotension are the most frequent adverse events with ABs [20]. The latter likely contributes to a 14% increase in the risk of falling and 16% increase in risk of sustaining a fracture in older men within 90 days of commencing an AB [21]. Caution should be exercised when considering ABs in those with cardiovascular disease or on vasodilatory medications. A specific adverse event associated with ABs is intraoperative floppy iris syndrome (IRIS) which can increase the risk of operative complications at the time of cataract surgery [22]. It is therefore important that patients, urologists and ophthalmologists are aware of the risks to ensure the AB is stopped in advance of surgery. ABs can cause ejaculatory dysfunction (OR 5.88) with more selective A1a receptor blockers such as tamsulosin and silodosin having a greater risk of this (OR 8.58 and 32.5, respectively) than non-selective ABs (e.g., terazosin OR 1.78) [23]. The importance of this factor to each individual patient may influence the choice of AB. However, ABs may result in a modest improvement in erectile dysfunction and do not appear to affect libido [24]. ABs do not impact on prostate size and symptoms typically progress with one study reporting that 14.2% experienced clinical progression at 4 years with 7.8% undergoing surgery for BPO [25].

### 5.2. 5-Alpha Reductase

The 5-alpha reductase (5AR) enzyme converts testosterone to the more potent dihydrotesterone (DHT) which mediates androgen effects on the prostate, with the 5AR type 2 isoform being predominately expressed in the prostate [26]. Finasteride inhibits the type 2 5AR and dutasteride additionally inhibits type 1 5AR [27,28]. Finasteride and dutasteride result in an approximately 70% and 95% reduction in serum DHT, respectively [27]. The significance of this is that prostatic growth is androgen dependent and reduced levels of DHT contribute to apoptosis of prostate epithelial cells [28]. Over a period of 6–12 months’ treatment with 5AR inhibitor (5ARI) the prostate reduces in size by approximately 20%, with a 50% fall in PSA [28,29]. An important difference from ABs is the slower onset of action and patients should be counselled regarding this to manage expectations.

An additional action of finasteride is the suppression of prostatic vascular endothelial growth factor (VEGF) and therefore it may be used in men with recurrent haematuria secondary to prostatic bleeding after other causes have been eliminated [30,31]. Zhu et al. reported that the administration of finasteride in the pre-operative period prior to transurethral prostate surgery was associated with significantly less intraoperative blood loss [32].

The improvement in LUTS secondary to use of 5ARIs is durable and associated with a reduced risk of progression including AUR and BPO surgery, and flow rates have been sustained for 6 years in finasteride and 4 years in dutasteride [33]. Adverse events associated with 5-ARIs are predominately related to their hormonal function with reduced libido, erectile dysfunction and ejaculatory dysfunction [26].

### 5.3. Phosphodiesterase 5 Inhibitors

Phosphodiesterase 5 inhibitors (PDE5Is) can be used in LUTS with or without concurrent erectile dysfunction [9]. They act to increase the intracellular concentration of cyclic guanosine monophosphate (cGMP), and hence reduce detrusor and prostatic smooth muscle tone [34]. Although several different PDE5Is in differing doses have been investigated in men with LUTS, only tadalafil 5 mg OD is currently licensed for this in Europe [9]. A large meta-analysis of 3973 patients comparing tadalafil 5 mg with placebo in the treatment of men with LUTS suggestive of BPO reported significant improvements in both IPSS and International Index of Erectile Function (IIEF) scores [35]. A separate, older meta-analysis also found significant improvements in both IPSS and IIEF scores but no difference in Q_max_ when comparing PDE5Is with placebo [36].

Adverse effects reported by patients taking PDE5Is include flushing, gastroesophageal reflux, headache and back pain [36]. Wang et al. reported that overall tadalafil 5 mg OD was well tolerated although the rate of discontinuation secondary to adverse events was 79% higher compared to the placebo group [35]. There is limited data regarding the long-term efficacy and tolerability of tadalafil 5 mg once daily with most studies to date having follow-up of up to 12 weeks [35,36]. PDE5Is are contraindicated in those concurrently using nitrates and those with extensive cardiac disease, and hence a full medical and drug history should be undertaken prior to prescribing.

### 5.4. Other Medications

Muscarinic-receptor antagonists (MRAs) (e.g., tolterodine or solifenacin) are licensed for overactive bladder (OAB) and storage symptoms, whilst BPO typically results in voiding symptoms predominately [9]. Antimuscarinics can be associated with an increased PVR and therefore should be used with care in men with concurrent BPO. One study reported a significant rise in PVR when tolterodine was used in the presence of mild-to-moderate bladder outflow obstruction (BOO) (49 mL vs. 16 mL) although this did not result in an increased rate of AUR [37]. Nonetheless, EAU guidelines recommend avoiding antimuscarinics to treat overactive bladder medications in men with a PVR of >150 mL [9]. The most common adverse effects association with MRA include dry mouth, dyspepsia, constipation and micturition difficulties [37].

Beta-3 agonists (B3As) (e.g., mirabegron) are similarly used in patients with OAB/storage symptoms and are often tolerated better than antimuscarinic medication [38]. Liao et al. investigated the efficacy and safety of mirabegron monotherapy in men with OAB with or without concurrent BOO, and found that both groups were similarly satisfied in terms of QoL and patient perception of symptoms [39]. However, only those without BOO had significantly improved IPSS values and the BOO group had an increased incidence of adverse effects. Adverse effects seen with B3A include dry mouth, constipation, dizziness and hypertension with mirabegron consequently being contraindicated in patients with uncontrolled hypertension [9,39].

### 5.5. Combination Therapy

Several different combination therapies have been investigated between different classes of drugs with the aim of having a greater overall improvement of symptoms when compared with monotherapy. However, it should be acknowledged that adverse effects from both drugs are seen when using combined treatments.

Combination therapy with a 5ARI and an AB has been shown to be superior to monotherapy of either agent at reducing risk of clinical progression and at symptom relief at four years [25]. This combination therapy can provide immediate relief of symptoms with the AB and more durable symptom improvement with the slow-onset 5ARI. Both the AUA and EAU recommend combination therapy in men with moderate-to-severe LUTS and enlarged prostates (>30–40 mL) [9,10].

Combination therapy of AB with either a MRA or a B3A are recommended if storage symptoms have been insufficiently treated with monotherapy [9]. A meta-analysis found that combination treatment of AB with an anti-muscarinic resulted in improvement of both storage symptoms and QoL without a deterioration in voiding function [40]. The MATCH RCT compared mirabegron and tamsulosin with placebo and tamsulosin for 12 weeks finding reduced mean number of daily voids and a small improvement in IPSS in the former [41].

AUA guidelines recommend against combination therapy of tadalafil 5 mg and AB for treatment of LUTS secondary to BPH [10]. However, a more recent meta-analysis found significant improvements in IPSS, QoL score, IIEF and Q_max_ with combination therapy (tamsulosin and tadalafil) compared to tamsulosin monotherapy [42]. There was a higher incidence of adverse effects in the combination group with pain being most commonly reported; however, there was no significant difference in the discontinuation rates between the groups.

### 5.6. Supplements

A wide array of different supplements and herbal preparations have been used to improve LUTS; however, there is only limited evidence to support the use of many of these. Saw palmetto (Serenoa repens) is the most widely studied herbal supplement, with hexane-extracted Serenoa repens (HESr) (Permixon^®^) recently being included in EAU guidelines as a weak recommendation [9]. AUA guidelines do not currently recommend any supplements or herbal preparations. Permixon^®^ has been shown to inhibit smooth muscle contraction in both the prostate and detrusor as well as inhibiting prostate stromal cell growth [43].

Different brands and preparations are not deemed to be equivalent, and this can make comparisons more challenging. A 2018 meta-analysis of trials looking specifically at the Permixon^®^ brand of HESr reported improvement in Q_max_ by 2.75 mL/s and fewer voids per night when compared to placebo [44]. HESr had no significant sexual dysfunction associated with its use and overall was well tolerated with gastrointestinal disorders being the most common side effect in 3.8% of men [44]. A more recent meta-analysis, although not looking specifically at HESr, compared Serenoa repens with placebo reported a minimal improvement in IPSS associated with Serenoa repens at 3–6 months although this was not deemed to be of clinical significance [45]. Ultimately, current evidence suggests that any improvement in LUTS due to BPO from taking HESr is likely to be modest at best, although the side-effect profile is favourable regarding sexual function and some men may choose to consider HESr provided they are prepared to accept the limited efficacy [9]. Combination therapy with HESr and AB has some evidence to support improved outcomes but few of these studies are RCTs [46].

## 6. Surgical Management

Transurethral resection of prostate (TURP) has long been the surgical standard of treatment for BPO due to a long history of use and detailed understanding regarding risk profile and long-term implications. However, a multitude of different surgical techniques have emerged in recent years and often these are compared to TURP (Table 1). Surgery is indicated in patients with moderate-to-severe LUTS, particularly for voiding symptoms and in those who have failed to achieve adequate symptom relief with conservative or medical treatment.

### 6.1. Transurethral Resection of Prostate

TURP is the most established surgical technique in BPO management with several decades of experience and consequently has a broad volume of data with excellent awareness of risks and long-term outcomes [47]. TURP can be performed with either monopolar (M-TURP) energy requiring a glycine irrigation fluid or bipolar (Bi-TURP) energy which can use 0.9% sodium chloride for irrigation. The latter can reduce the incidence of transurethral resection (TUR) syndrome that can arise from intraoperative absorption of hypotonic irrigation fluid during M-TURP, and subsequent dilutional hyponatraemia and fluid overload [6].

TURP results in significant improvement in IPSS, Q_max_ rate, QoL score and PVR [48,49]. Outcomes from M-TURP are durable with a 1–2% annual rate of repeat prostatic surgery often reported, supported by an 8.3% rate of redo-TURP reported at 8 years in a study analysing Austrian national registry data [50]. A large Cochrane review concluded that both M-TURP and Bi-TURP improve urological symptoms to a similar degree and, although Cornu et al. reported a small difference in Q_max_ at 12 months favouring Bi-TURP, this may not be clinically significant [48,51]. However, the safety profile of Bi-TURP is favourable with reduced incidence of TUR syndrome and lower blood transfusion rates. TURP additionally provides tissue for histological analysis which can detect incidental prostate adenocarcinoma, which may or may not be clinically significant. A large prospective multi-centre study reported a 9.8% rate of incidental prostate cancer [49].

Austrian national data shows a 0.1% risk of perioperative mortality associated with TURP at 30 days which represents a 20% reduction when compared with the previous decade [50]. A large multi-centre German prospective study from 2002 to 2003 reported an overall morbidity rate of 11.1% with urinary retention (5.8%), urinary tract infection (3.6%), blood transfusion due to peri- or post-operative bleeding (2.9%) and TUR syndrome (1.4%) [49]. Long-term complications include urinary incontinence, bladder neck stenosis, urethral stricture, erectile dysfunction and retrograde ejaculation [6].

EAU guidelines recommend offering M-TURP or Bi-TURP for moderate-to-severe LUTS with a prostate volume of 30–80 mL. The rationale behind the upper limit is based on the increased side effect profile associated with longer operative times and 80 mL was deemed as the upper limit of what could achievably be resected within a 90-min maximum recommended operative time [52].

A disadvantage associated with TURP is the longer length of stay due to the need for post-operative three-way catheterisation and continuous bladder irrigation with a mean length of stay of 3.6 days in 2012 [53]. However, some centres are now performing day-case TURP by discharging patients with a catheter in situ and a plan to return to clinic in 48 h for catheter removal [54].

### 6.2. Open Simple Prostatectomy

Open simple prostatectomy (OSP) to remove obstructive prostatic adenoma via the transvesical approach (Freyer procedure) or anterior prostatic capsule (Millins procedure) have historically been the treatment of choice for large prostates (>80 mL). Studies have demonstrated comparable improvement in IPSS score including QoL, Q_max_ and reduction in PVR for both HoLEP and Bi-TURP [55]. A reintervention rate of 6% was reported at 5 years using Austrian national registry data [50]. Although significantly improved over the last 20–30 years, there is a mortality rate associated with OSP of approximately 0.2% at 30 days [50].

Whilst OSP offers similar improvement in LUTS and other objective outcomes when compared with HoLEP, it is more invasive and associated with a higher risk of peri-procedural complications, including mortality with a prolonged hospital admission [55]. OSP has a significantly higher blood transfusion rate than HoLEP and long-term complications of OSP include urethral stricture, bladder neck contracture and urinary incontinence [56]. The advantages of transurethral procedures have resulted in a decline in the number of OSP performed annually, which is having implications for training of residents and thus limiting the number of urologists competent in safely performing the procedure [57,58].

### 6.3. Minimally Invasive Simple Prostatectomy

The perioperative risks associated with OSP and developments in laparoscopic and robotic surgery have contributed to the development of minimally invasive simple prostatectomy (MISP). Laparoscopic simple prostatectomy (LSP) was first described and, as with many other urological procedures, this has transitioned to robot-assisted simple prostatectomy (RASP) more recently.

A meta-analysis comparing MISP with OSP found no significant differences in Qmax, IPSS and PVR, but MISP was associated with reduced length of hospital stay, blood loss and length of catheterisation with a lower complication rate [59]. A RCT of 110 patients with prostate volume >120 mL compared RASP, LSP and HoLEP, and found no significant differences in perioperative or functional outcomes, although MISP had longer length of hospitalisation than HoLEP [60]. The literature to date suggests MISP is likely just as efficacious as open prostatectomy but with fewer complications and shorter length of stay. Large prospective studies are required to evaluate long-term outcomes alongside cost analysis and the learning curve of these techniques.

### 6.4. Laser

Various lasers have been introduced into the field of endourology and these have also been utilised in treatment of BPO. Holmium, Greenlight, Thulium and diode lasers have been implemented and they have been used for vaporisation or enucleation of prostatic tissue [61].

A. Photoselective Vaporisation of Prostate (PVP)

The 532 nm Potassium-Titanyl-Phosphate (KTP) or Lithium triborate (LBO) Greenlight laser uses a side-on-laser and the short wavelength results in increased absorption by oxyhaemoglobin and tissue vaporisation with coagulation of tissue [61]. The 80 W, 120 W and the most recent 180 W GreenLight XPS system have been developed since 2005. The Goliath multi-centre RCT of 281 patients demonstrated non-inferiority of Greenlight XPS compared with TURP with similar IPSS and Q_max_ scores [62]. The rates of late complication and need for retreatment at two years was also equivalent in both the PVP and TURP groups [63]. Lichy et al. reported a significant improvement in the rate of perioperative complications over the 9 year period from 2011 and 2019 as operator experience increased [64].

A more recent RCT compared Greenlight with transurethral resection in saline (TURis) and HoLEP for larger prostates (80–150 mL) with a median size of 105 mL, and all resulted in improvement in IPSS with TURis having a higher rate of intraoperative complications and longer length of stay [65]. The procedure length was longer in Greenlight with a higher rate of redo surgery for both Greenlight and TUR at 3 years compared with HoLEP [65]. A long-term review of the Finnish national registry showed that there was a lower risk of re-operation for bleeding than TURP and therefore may be more suitable for patients on anticoagulation [66]. However, the overall reoperation rate at 12 years was higher for PVP than TURP, at 23.5% and 17.8%, respectively [66]. The authors postulate that this may be a result of older generation lasers and in fact the newer 180 W laser system may produce more favourable re-operation rates.

An important consideration in relation to PVP is the cost-effectiveness as, unlike Holmium and Thulium laser systems which can be multi-purpose for BPO and stone use, the greenlight laser is only licensed to treat BPO, therefore necessitating centres to purchase a second laser for stone management.

B. Holmium Laser Enucleation of Prostate (HoLEP)

The Holmium:YAG laser is a 2140 nm wavelength pulsed laser that is absorbed by water and water containing tissues with tissue coagulation and necrosis limited to 3–4 mm, and thus enabling haemostasis [61]. A meta-analysis comparing M-TURP and HoLEP reported that HoLEP is associated with similar short-term efficacy but significantly fewer complications, reduced need for blood transfusion, shorter catheterisation and shorter duration of hospital stay [48]. HoLEP was associated with a longer operative duration but at 1 year there were significant differences for IPSS, PVR and Q_max_ favouring HoLEP.

Long-term follow-up with a median of 126 months post HoLEP have demonstrated the durability of HoLEP with Q_max_ 16 mL/s, PVR 10 mL and IPSS 5 [67]; 4.7% underwent redo surgery for bladder neck contracture or urethral stricture, and 5.7% reported incontinence [67]. A further meta-analysis suggests that HoLEP performs favourably when compared with other techniques including Bi-TURP and bipolar enucleation of prostate (BPEP) in the treatment of large volume prostates (>80 mL) [68].

One of the major challenges with HoLEP is the relatively long learning curve and a structured mentoring programme can ensure safety during the learning curve whilst achieving good surgical outcomes [69]. Pulse modulation technology with the second-generation Moses platform has been designed to improve tissue enucleation and vessel haemostasis. In a study comparing standard HoLEP, Moses-HoLEP was associated with equivalent outcomes for IPSS, Q_max_, PVR and complications [70]. Although overall operative duration was similar, Moses-HoLEP had significantly faster mean haemostasis time and achieved a 69.4% rate of same-day discharge [70].

C. Thulium Laser

The Thulium:YAG laser has a wavelength of 1940–2013 nm with a continuous waveform and has been used in different applications for treatment of BPO including vaporesection (ThuVARP), enucleation (ThuLEP) or vapoenucleation (ThuVEP) [61].

A meta-analysis found that ThuLEP was non-inferior to TURP regarding both operative and functional outcomes, and had superior outcomes for shorter catheter duration and hospital stay [71]. A meta-analysis comparing ThuLEP with HoLEP reported comparable improvement in symptoms and voiding function for up to 18 months post-operatively [72]. Operative time and hospital duration were also equivalent, but ThuLEP was associated with slightly lower blood loss and lower rates of transient incontinence than HoLEP [72]. ThuVEP has a smaller evidence base consisting predominately of prospective single-centre case series with Chang et al. reporting significant improvements in IPSS and Q_max_ from baseline to 12 months with an overall complication rate of 20.7% [73].

### 6.5. Bipolar Transurethral Enucleation of Prostate

Bipolar transurethral enucleation of the prostate (BPEP) uses bipolar energy to enucleate the obstructive prostatic adenoma followed by either resection or morcellation of the enucleated adenoma. A meta-analysis reported shorter length of stay, reduced post-operative complications with lower haemoglobin drop and reduced reintervention rate for BPEP when compared to Bi-TURP [74]. Functional outcomes were broadly similar, although BPEP was associated with significantly lower IPSS at 6 months compared to B-TURP but no difference at other time-points [74]. BPEP has been demonstrated to have similar efficacy to OSP for larger prostates with similar operative duration and lower rate of blood transfusion compared to OSP [55]. A meta-analysis comparing BPEP with HoLEP reported the latter was associated with shorter operative duration, reduced haemoglobin loss and reduced length of hospital stay [68].

## 7. Alternative Modalities

### 7.1. Aquablation

Aquablation of the prostate uses the robotic Aquabeam system with ultrasound guidance to enable targeted ablation of the prostatic parenchyma by hydro-dissection with high-velocity saline [75,76]. Haemostasis is variably achieved with a three-way catheter and irrigation or diathermy [76]. A RCT comparing aquablation with TURP for men with prostates 30–80 mL and moderate-to-severe LUTS secondary to BPO showed substantial improvements in IPSS at 6 months thus satisfying the non-inferiority hypothesis [77]. The anejaculation rate was lower in the aquablation group compared to the TURP group (10% vs. 36%). Operative times were similar for Aquablation and TURP (33 vs. 36 min) but aquablation had a significantly lower resection time of 4 vs. 27 min [77]. The 3-year follow-up demonstrated sustained improvement in IPSS in both aquablation and TURP groups with mean improvement of 14.4 and 13.9 points, respectively, and Q_max_ improvements of 11.6 and 8.2 mL/s, respectively [78]. The three year retreatment rate was 4.3% in the aquablation group and 1.5% in the TURP group [78]. A further study investigated the role of aquablation in larger prostates (80–150 mL) in 101 men, with significant improvements in IPSS, QoL, Qmax and PVR that was sustained for 3 years with a 3% rate of repeat surgery for LUTS [79]. A recent systematic review corroborates the findings that both IPSS and Q_max_ are significantly improved from baseline following aquablation up to one year whilst appearing to preserve sexual function at 3 months, although the meta-analysis was limited by the extent of the heterogeneity between studies [80]. Post-operative bleeding necessitating a return to theatre or blood transfusion have been reported with Bach et al. reporting a 7.9% rate of return to theatre for haemostasis, a 2 g/dL drop in haemoglobin level prior to discharge and a 2.7% transfusion rate [81].

### 7.2. Rezūm™

Water ablative therapy with sterile water vapor injection into the prostate with subsequent prostatic tissue necrosis using the Rezūm system has been available since 2015 in the USA and 2018 in the UK [82]. A transurethral device is used to deliver steam treatment, typically with one to three treatments to each lateral lobe, and one or two to the median lobe if present [82]. It can be performed under sedation and as a day case procedure [82]. An early RCT in 2016 comparing Rezūm with sham treatment found a significant improvement in IPSS with improved Qmax at 3 and 12 months [83]. An advantage of Rezūm is that both erectile and ejaculatory function are typically preserved with de novo rates of erectile dysfunction ranging from 0–3.1% in a systematic review [83,84]. A more recent systematic review suggests that improvement in IPSS is resilient as far as 5 years with a surgical retreatment rate of 4.4–7.5% at 5 years [84].

Although an upper limit for prostate volume of 80 mL is typically advised, success has been reported in those with larger prostates and also in those with previous urinary retention [85].

### 7.3. Prostate Artery Embolisation (PAE)

PAE is a minimally invasive procedure which can be performed under local anaesthetic by interventional radiologists (IR). Access is established through the femoral or radial arteries and digital subtraction angiography is used to delineate the prostatic arterial supply to allow selective embolisation, of which several techniques have been described in the literature. In an RCT comparing PAE with a sham procedure in men with symptoms refractory to medical treatment, a significant improvement in IPSS was seen in the PAE group compared to the sham group [86]. However, when compared to TURP, PAE is less efficacious with regards to IPSS score, Q_max_, prostate volume and PVR [87]. However, a further meta-analysis has reported that PAE is associated with a lower risk of sexual dysfunction than TURP (OR 0.24) [88]. Initial prostate size and percentage reduction in prostate volume at 3 months have been identified as important factors in determining the success of achieving symptomatic relief with PAE [89].

PAE remains a valid option for some patients particularly those who are prepared to accept a reduced improvement in urinary symptoms compared with TURP but lower risk of sexual dysfunction. Similarly, it may also be an option for frail patients in which a general anaesthetic procedure is deemed to be high risk [88]. It should be noted that AUA currently recommends PAE should not be used outside of clinical trials due to uncertainty regarding the benefits [11]. Nonetheless, where it is used, it is essential for multidisciplinary discussions between the urology and IR teams to identify suitable patients and ensure appropriate post-procedural follow-up.

### 7.4. Prostatic Urethral Lift (PUL)

PUL (Urolift^®^) is a minimally invasive procedure that involves the placement of trans-prostatic suture-based implants under cystoscopic guidance to compress obstructing lateral lobes and therefore open the prostatic urethra [90]. PUL was not designed to treat obstructive median lobes and therefore this should be excluded prior to PUL which can necessitate an additional procedure in the form of a flexible cystourethroscopy. However, a recent study suggests that treatment of obstructive median lobe with Urolift^®^ can result in significant improvement in LUTS [91]. PUL is associated with a significant improvement in IPSS, QoL and Q_max_ [92,93].

Durability of results has been confirmed by one study comparing PUL with sham treatment with IPSS, Qmax and QoL improvement rates of 36%, 50% and 44% from baseline at 5 years, respectively [93]. An RCT comparing PUL with TURP found that the improvement in IPSS and Q_max_ was significantly greater in the TURP group whilst QoL was equivalent [94]. Most studies to date have investigated the role of PUL on prostate sized 30–80 mL and this is reflected in the current guidelines recommending this cut-off. However, Shah et al. compared outcomes of patients undergoing PUL with prostate volume <80 g and >80 g with a median prostate size of 112 g in the latter group [95]. They found that more implants were needed in the larger prostate group, but no differences were seen in IPSS or need for additional procedures between the two groups, although the sample size was small.

PUL is generally well tolerated with dysuria (9%), pelvic pain (6%), haematuria (4%) and urge urinary incontinence (3%) being most commonly reported and mostly resolving within four weeks of the procedure [93]. PUL is associated with preservation of sexual function and may result in a small improvement in IIEF scores [92,94]. The annual rate of surgical reintervention has been reported as 6% annually, with TURP/laser prostatectomy (51.0%), repeat PUL (32.7%) and explant of device (19.6%) being most common [96].

As Urolift can be performed as a day-case procedure, often under local anaesthetic, this can be more cost-effective than other surgical options for BPO with an estimated cost-saving of £981 per person in the United Kingdom compared with Bi-TURP [97].

## 8. Future Modalities

### 8.1. iTind

iTind is a temporary implantable nitinol device that has three struts at the 12, 5 and 7 o’clock positions with an anchoring leaflet and is deployed under direct visualisation within the prostatic urethra [98]. The device exerts continuous pressure and therefore creates ischaemic necrosis resulting in remodelling of the prostatic urethra and bladder neck, and is removed after 5 days via cystoscopy. A RCT comparing iTind with sham procedure reported that, at 12 months, the iTind group had a 9.25 point reduction in IPSS, 3.52 mL/s increase in Q_max_ and 1.9 point improvement in QoL score [99]. The procedure was typically well tolerated with mild, transient adverse effects reported and no de novo ejaculatory or erectile dysfunction. Further studies are required to compare iTind with other treatment modalities, and to establish the long-term outcomes and need for re-treatment.

### 8.2. Optilume

The Optilume BPH catheter system is a drug-coated device that has a dual function with balloon dilatation to achieve anterior commissurotomy to open the urethral lumen followed by delivery of paclitaxel to maintain the urethral patency [100]. The PINNACLE study is an RCT that compared Optilume BPH system with sham procedure [101]. Significant improvements from baselines were seen in IPSS, QoL and Q_max_ with a 49% improvement in IPSS at 1 year. A total of five serious adverse events occurred, four cases of post-procedural haematuria requiring cystoscopic management or extended observation, and one urethral false passage necessitating prolonged catheterisation. The most common adverse events were haematuria (40%), UTI (14%), dysuria (9.2%) and urge/mixed incontinence (8.2%), and these typically resolved within four weeks. Although IIEF scores were slightly improved post-procedure, 4.1% reported de novo ejaculatory dysfunction in the Optilume group.

## 9. Cost-Effectiveness

Comparisons between the cost-effectiveness of different medical and surgical treatments for BPO are complicated by heterogeneity in methodology between studies, and variability in costs and tariff systems between countries. A cost minimisation analysis comparing medical therapy with TURP across five different European countries reported substantial variation between countries in medication costs and TURP treatment tariffs [102]. Consequently, the duration of treatment for which medical therapies were more cost-effective than TURP ranged from 2.9 to 70.4 years [102]. A cost–utility analysis in the US suggested that HoLEP was more cost-effective at 5 years when considering both overall costs and quality of life compared to B-TURP, PUL, water ablative therapy and OSP [103].

## 10. Conclusions

BPO is a common urological condition that has a significant impact on quality of life and economic burden globally, and is likely to become increasingly prevalent with an ageing population. Whilst TURP and medical management still have important roles in the management of BPO, a plethora of alternatives are now available including laser enucleation and minimally invasive techniques. Selecting the most appropriate treatment modality will depend on the individual patient preferences, availability of resources, cost, anatomical factors and the goals of treatment.

## Figures and Tables

**Table 1:** Summary of selected surgical management options in BPO.

Modality	Advantages	Disadvantages	AUA Recommendations	EAU Recommendations
**Transurethral Resection of Prostate (TURP)**	Effective at improving urinary symptoms rapidly. Widely available. Large body of evidence with good understanding of safety profile.	Procedural risks include anejaculation and erectile dysfunction. TUR syndrome may occur with longer procedures, which can be reduced with bipolar approach.	Should be offered as a treatment option. Monopolar or bipolar approaches may be used depending on expertise.	To treat moderate-to-severe LUTS in men with prostate size 30–80 mL.
**Photoselective Vaporisation of Prostate (PVP)—‘Greenlight laser’**	Improved intraoperative haemostasis compared to TURP. May be safe in men receiving antiplatelet or anticoagulant therapy. Reduced length of hospital stay.	Longer operative duration compared to TURP Cost associated with requirement for a dedicated laser for Greenlight procedures.	120 W or 180 W platforms should be offered to treat LUTS/BPH.	Offer 80 W KTP, 120 W LBO or 180 W LBO laser vaporisation of the prostate in moderate-to-severe LUTS in men of prostate volume 30–80 mL as an alternative to TURP.
**Holmium Laser Enucleation of Prostate (HoLEP)**	Similar efficacy to TURP in the mid- and long-term. Suitable for larger prostates (>80 mL). Improved perioperative safety profile compared to TURP.	Longer learning curve than TURP. Longer operative duration compared to TURP. Costs associated with acquisition of equipment.	Should be considered as a prostate size-independent option for treatment of LUTS/BPH.	Offer for moderate-to-severe LUTS in men as an alternative to TURP or OP.
**Thulium Laser**	Effective in relieving LUTS in both moderate and large prostates. May be safe in those receiving antiplatelet or anticoagulant therapy.	Fewer high-quality studies compared to HoLEP or TURP. Costs associated with laser acquisition.	Should be considered as a prostate size-independent option for treatment of LUTS/BPH.	Offer to men with moderate-to-severe LUTS as an alternative to HoLEP/TURP/B-TUEP.
**Open Prostatectomy (OSP)**	Effective and durable procedure to treat BPO. Suitable for large prostates (>80 mL).	Most invasive modality. Transfusion rate ~10%. Mortality rate 0.2% at 30 days. Prolonged length of hospital admission.	Should be considered by clinicians with appropriate expertise on patients with large or very large prostates.	Offer for moderate-to-severe LUTS if prostate volume >80 mL and HoLEP/B-TUEP unavailable.
**Laparoscopic/Robotic Prostatectomy**	Lower complication rate and shorter length of hospital stay compared to OSP. Data suggest comparable functional outcomes to OSP.	Lack of high-quality data to support use. Longer learning curve.	Should be considered by clinicians with appropriate expertise on patients with large (80–150 g) or very large prostates (>150 g).	No current recommendation due to lack of RCTs.
**Aquablation**	Efficacy comparable to TURP for improving LUTS in BPO. May also be effective in men with larger prostates (80–150 mL). Short learning curve. Lower risk of sexual dysfunction compared to TURP.	Some concerns regarding perioperative bleeding. General anaesthesia and inpatient admission required.	May be offered as a treatment option for those with LUTS/BPH and a prostate volume of 30–80 mL.	Offer as an alternative to TURP for men with moderate-to-severe LUTS and a prostate volume of 30–80 mL. Inform patients of risk of bleeding and limited long-term follow-up data.
**Prostate Artery Embolisation (PAE)**	Minimally invasive procedure that can be performed under local anaesthetic.	Impaired efficacy in relieving LUTS compared to TURP. Large radiation dose delivered to patient.	Not currently recommended outside of clinical trials as the benefit over risk remains clear.	Weak recommendation to offer to men with moderate-to-severe LUTS that accept less favourable outcomes to TURP for a less invasive procedure. Only perform in units where work-up and follow-up are performed collaboratively with urologists and interventional radiologists.
**Water Ablative Therapy (Rezūm)**	Favourable safety profile. Sexual function likely to be preserved. Short learning curve.	Limited comparisons against reference technique (e.g., TURP).	Should be considered as a treatment option for LUTS if prostate volume 30–80 mL. May be offered to those who desire preservation of erectile and ejaculatory function.	No recommendation made as randomisation against a reference technique is required.
**Prostatic Urethral Lift (PUL)—Urolift**	Relatively short learning curve. May be performed under local or general anaesthetic. Erectile and ejaculatory function preserved.	Not recommended for those with an obstructing middle lobe and verifying this prior to procedure potentially adds to cost. Limited data on long-term durability and need for repeat intervention.	Consider for patients with LUTS providing prostate volume 30–80 mL and verified absence of obstructing middle lobe. May be offered to those who desire preservation of erectile and ejaculatory function.	Offer to men with LUTS with prostate <70 mL and no middle lobe that wish to preserve ejaculatory function.

## Data Availability

Not applicable.
